# Temporal Variability of Cortical Gyral-Sulcal Resting State Functional Activity Correlates With Fluid Intelligence

**DOI:** 10.3389/fncir.2019.00036

**Published:** 2019-05-15

**Authors:** Shimin Yang, Zhongbo Zhao, Han Cui, Tuo Zhang, Lin Zhao, Zhibin He, Huan Liu, Lei Guo, Tianming Liu, Benjamin Becker, Keith M. Kendrick, Xi Jiang

**Affiliations:** ^1^The Clinical Hospital of Chengdu Brain Science Institute, MOE Key Lab for Neuroinformation, School of Life Science and Technology, University of Electronic Science and Technology of China, Chengdu, China; ^2^School of Automation, Northwestern Polytechnical University, Xi’an, China; ^3^Department of Computer Science, Bioimaging Research Center, The University of Georgia, Athens, GA, United States

**Keywords:** functional activity, temporal variability, cortical folding, gyri and sulci, resting state fMRI, fluid intelligence

## Abstract

The human cerebral cortex is highly convoluted as convex gyri and concave sulci. In the past decades, extensive studies have consistently revealed substantial differences between gyri and sulci in terms of genetics, anatomy, morphology, axonal fiber connections, and function. Although interesting findings have been reported to date to elucidate the functional difference between gyri and sulci, the temporal variability of functional activity, which could explain individual differences in learning and higher-order cognitive functions, and as well as differences in gyri and sulci, remains to be explored. The present study explored the temporal variability of cortical gyral-sulcal resting state functional activity and its association with fluid intelligence measures on the Human Connectome Project dataset. We found that the temporal variance of resting state fMRI BOLD signal was significantly larger in gyri than in sulci. We also found that the temporal variability of certain regions including middle frontal cortex, inferior parietal lobe and visual cortex was positively associated with fluid intelligence. Moreover, those regions were predominately located in gyri rather than in sulci. This study reports initial evidence for temporal variability difference of functional activity between gyri and sulci, and its association with fluid intelligence measures, and thus provides novel insights to understand the mechanism and functional relevance of gyri and sulci.

## Introduction

One of the most prominent organization principles of the human cerebral cortex lies in its highly convoluted folding patterns which are composed of convex gyri and concave sulci (Barron, 1950; Rakic, 1988; Zilles et al., 1988; Welker, 1990). The past decades have witnessed a variety of hypotheses regarding the complex gyrification process, including cortex area increase and compact wiring (Zilles et al., 2013), genetic regulation (Rakic, 2004), differential laminar growth (Richman et al., 1975), and axonal fiber tension (Van Essen, 1997). Although the precise mechanisms of gyrification process are still under debate, a growing number of studies suggests substantial differences between gyri and sulci in terms of genetics (Stahl et al., 2013; Zeng et al., 2015), anatomy (Fischl and Dale, 2000; Li et al., 2015), morphology (Magnotta et al., 1999; Hilgetag and Barbas, 2005), and axonal fiber connections (Van Essen, 1997; Nie et al., 2012; Takahashi et al., 2012; Chen et al., 2013; Deng et al., 2014; Zhang et al., 2014; Li et al., 2015; Ge et al., 2018). For instance, cortical thickness is significantly larger in gyri than in sulci in both human developing infant (Li et al., 2015) and adult brains (Fischl and Dale, 2000). The axonal connectivity and gene expression patterns are significantly different between cerebellum gyri and sulci of rodent brains (Zeng et al., 2015). The diffusion weighted imaging derived axonal fibers concentrate significantly more on gyri than on sulci, which is developmentally and evolutionarily consistent across human fetus, human adult, chimpanzee, and macaque brains (Nie et al., 2012; Takahashi et al., 2012), and was supported by histology and dissection studies (Xu et al., 2010; Budde and Annese, 2013). The diffusion weighted imaging derived axonal fiber connection strength is strong between gyral–gyral regions, weak between sulcal–sulcal regions, and moderate between gyral-sulcal regions (Deng et al., 2014). Moreover, previous studies have reported that the morphological feature of gyri and sulci changes during aging (Magnotta et al., 1999) and development-related psychiatric disorders such as schizophrenia (White et al., 2003), reflecting a potential of gyral-sulcal indices as a biomarker for developmental and aging related disorders.

Given the close relationship between brain structure and function (Passingham et al., 2002; Zhang et al., 2011) and with the development of advanced *in vivo* functional neuroimaging such as functional MRI (fMRI) (Biswal et al., 1995; Logothetis, 2008; Friston, 2009), the functional characteristics of gyri and sulci has gained increasing interests in recent years (Deng et al., 2014; Jiang et al., 2015, 2018a,b; Liu et al., 2017, 2018; Zhang et al., 2018). A multi-modal diffusion tensor imaging (DTI) and fMRI study has reported that both structural fiber connectivity and functional connectivity are strong between gyral–gyral regions, weak between sulcal–sulcal regions, and moderate between gyral-sulcal regions in the whole-brain, suggesting that gyri represent a global functional hub and sulci a local function processing unit (Deng et al., 2014). The heterogeneous functional regions which are activated during multiple task conditions locate significantly more in gyri than in sulci under both temporal stationary (Jiang et al., 2015) and dynamics (Jiang et al., 2018a). The graph-theoretic characteristics of functional interaction (Liu et al., 2017) and functional signal reconstruction accuracy (Jiang et al., 2018b) are also different between gyri and sulci. In addition, recent studies using advanced deep learning methodologies have reported the frequency-specific pattern differences between gyri and sulci (Liu et al., 2018; Zhang et al., 2018).

In spite of these aforementioned interesting findings, the temporal variability of functional activity of gyri and sulci still remains to be explored. Instead of characterizing the static functional activity by simply averaging the fMRI blood-oxygen level-dependent (BOLD) signal, a growing number of recent studies have shown the temporal-varying dynamics of spontaneous neural activity within a single brain region as well as the functional connectivity/interaction between brain regions during both rest and task conditions (Gilbert and Sigman, 2007; Chang and Glover, 2010; Garrett et al., 2010; Protzner et al., 2010; Bassett et al., 2011, 2013, 2015; Smith et al., 2011; Calhoun et al., 2014; Li et al., 2014; Zhang et al., 2016; Vidaurre et al., 2017; Jiang et al., 2018a; Yuan et al., 2018). The temporal variability of functional activity reflected in neuroimaging fMRI signal could be related to the brain learning skill (Bassett et al., 2011, 2013, 2015; Zhang et al., 2016) and human intelligence (Saxe et al., 2018). Especially, fluid intelligence, as a measure of higher-order relational reasoning (Burgess et al., 2011), has been argued to be linked to specific functional outcomes and to variations in human neuronal structure and function (Duncan et al., 2000; Duncan, 2003, 2005). Previous studies have suggested that the higher temporal variability of brain functional activity might be closely linked with higher-order relational reasoning and learning (Bassett et al., 2011, 2013, 2015; Zhang et al., 2016; Saxe et al., 2018). Therefore, investigating the correlation between temporal variability in the resting state fMRI signal and fluid intelligence measures may allow to further determine whether intrinsic temporal variations in brain activity are related to individual variations in fluid intelligence. Taken together, investigating the temporal variability characteristics of functional activity as well as its associations with fluid intelligence on gyri and sulci could provide novel insights to understand the functional relevance of gyri and sulci.

To this end, the present study adopted 68 subjects with both resting state fMRI and fluid intelligence measures data in the publicly released Human Connectome Project (HCP) Q1 release (Barch et al., 2013; Smith et al., 2013) to test the hypothesized associations. We employed a previously evaluated approach to divide the fMRI BOLD signal into non-overlapping time segments in order to assess the temporal variability of functional activity on each gyral/sulcal region by means of calculating the variance of time series correlations among all time segments. To determine the behavioral relevance of the temporal variability we correlated the temporal variance with the available three fluid intelligence measures across subjects. Based on previous study (Duncan et al., 2000) reporting that frontal, parietal, and visual cortex were involved in intelligence-related cognitive tasks, we hypothesized that the temporal variability of functional activity in frontal, parietal, and visual cortex would positively correlate with the fluid intelligence measures. Furthermore, based on previous studies reporting the functional difference between gyri and sulci (Deng et al., 2014; Jiang et al., 2015, 2018a,b; Liu et al., 2017, 2018; Zhang et al., 2018), we hypothesized that the distribution of those brain regions would be different between gyri and sulci.

## Materials and Methods

### Participants, Image Acquisition, and Data Preprocessing

We adopted all 68 subjects in the publicly released Human Connectome Project (HCP) Q1 data (Barch et al., 2013; Smith et al., 2013; Van Essen et al., 2013) to test our hypotheses. There were 18 males and 50 females ranging from 22 to 35 years old. More demographic information was referred to Van Essen et al. (2013). The resting state fMRI (rsfMRI) BOLD signal was acquired using 3T “multiband” accelerated EPI when subjects were instructed to be relaxed, with eyes fixation on a white cross and not to fall asleep (Smith et al., 2013). The major acquisition parameters of rsfMRI data were as follows: 2 mm × 2 mm × 2 mm spatial resolution, TR = 0.72 s, TE = 33 ms, flip angle = 52°, 90 × 104 matrix, 72 slices, in-plane FOV = 208 mm × 180 mm, 1200 whole-brain volumes (14.4 min). The major preprocessing steps of rsfMRI data included skull removal, motion correction, slice time correction, and spatial smoothing (Smith et al., 2013).

Moreover, we adopted the preprocessed grayordinate rsfMRI data after the minimal preprocessing pipelines (Glasser et al., 2013) provided in the HCP datasets. The minimal preprocessing mainly included spatial artifacts and distortions removal, cortical surface generation, cross-subject registration to standard grayordinate space (Glasser et al., 2013). Specifically, the grayordinate space included both the cortical surface vertices and subcortical voxels of whole-brain gray matter in the MNI standard space. Each of the 60K grayordinate cortical surface vertices was associated with a set of geometric attributes and corresponding rsfMRI time series, and had correspondence across different subjects. Note that the grayordinate data had both high spatial and temporal resolution, thus can reliably differentiate gyri/sulci and map the fMRI signals on gyri/sulci. Therefore, it provided the prerequisite for the present study to investigate the temporal variability of cortical gyral-sulcal functional activity.

The fluid intelligence measures of the 68 subjects were also provided in the HCP dataset (Barch et al., 2013). The Penn Matrix Test was adopted to measure fluid intelligence via non-verbal reasoning using Form A of an abbreviated version of the Raven’s Progressive Matrices as detailed in Bilker et al. (2012). Three measures were finally provided for each subject: number of correct responses, median reaction time for correct responses, and total skipped items (items not presented due to maximum errors allowed reached in the test) (Barch et al., 2013).

### Cortical Surface Parcellation of Gyri and Sulci

**FIGURE 1:**
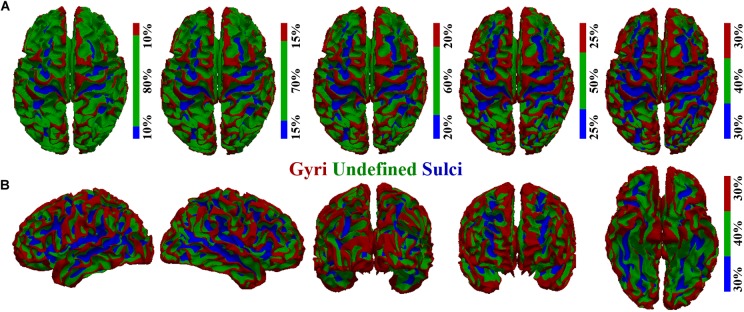
Cortical surface parcellation of gyri, sulci, and undefined. **(A)** The parcellated cortical surface when the convexity threshold value *q* equals 10, 15, 20, 25, and 30, respectively. **(B)** The parcellated cortical surface when *q* equals 30 from another five different views.

To investigate the potential difference of BOLD signal temporal variability between cortical gyri and sulci, we first performed cortical surface parcellation to classify the cortical vertices into gyri and sulci. The average convexity (i.e., “sulc” map in FreeSurfer) of each cortical vertex, defined as the signed distance of the movement during inflation with the surface normal (Fischl et al., 1999; Destrieux et al., 2010; Fischl, 2012), was adopted to classify gyri and sulci in line with previous studies (Jiang et al., 2015, 2018a,b; Liu et al., 2018; Zhang et al., 2018). A single vertex with higher average convexity value would be more likely to be classified as gyri and vice versa, resulting in the highest convexity values in the crown of gyri and lowest values in the fundi of sulci. Moreover, there were transitional or intermixed areas between gyri crown and sulci fundi. To avoid any ambiguity and to ensure the accuracy of gyri/sulci parcellation, we set a threshold value *q* for the convexity values of all cortical vertices in line with previous studies (Liu et al., 2018; Zhang et al., 2018). The *q*% vertices with highest convexity values would be classified as gyri, and the *q*% vertices with lowest convexity values would be classified as sulci. The remaining (100-2*q*)% transitional vertices between gyri and sulci would be classified as “undefined” (which means not defined as gyri or sulci). As a consequence, the cortical surface was parcellated into three parts: gyri, sulci, and undefined (Figure 1). We tested different threshold values *q* ranging from 10 to 30 as shown in Figure 1A,B shows the parcellated cortical surface from different views when *q* = 30. The rsfMRI BOLD signal was then extracted for each vertex on gyri/sulci/undefined region. It is noted that our following temporal variability analyses were applied to the spectrum of *q*s.

### Temporal Variability Assessment of Functional Activity on Cortical Gyri and Sulci

**FIGURE 2:**
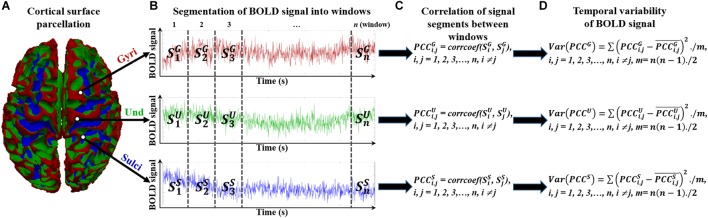
Temporal variability assessment of functional activity in cortical gyri, sulci, and undefined regions. **(A)** The parcellated cortical surface of gyri, sulci, and undefined regions. **(B)** Segmented *n* non-overlapping time windows of each BOLD signal in gyri, sulci, and undefined regions. **(C)** Assessment of correlation between any pair of signal segments in time windows. **(D)** Assessment of variance of all correlation values between time window pairs.

To assess the temporal variability of functional activity on cortical gyri and sulci, we divided the rsfMRI BOLD signal into non-overlapping windows in line with previous study (Zhang et al., 2016). The overlapping windows would introduce the common signal segments for two consecutive windows and could bias the correlation value of BOLD activity of the two consecutive windows. The detailed framework is presented in Figure 2. For a BOLD signal of gyri, sulci, or undefined region (Figure 2A), it was divided into *n* non-overlapping windows with size *l* (Figure 2B). We tested different window size *l* ranging from 25 to 200 to avoid arbitrary choice of window size. Within the time window *i*, the BOLD signal segment was annotated as $S_{i}^{G}$, $S_{i}^{S}$, and $S_{i}^{U}$ for gyral, sulcal, and undefined vertex, respectively (Figure 2B). For any pair of signal segments in time window *i* and *j* (*i, j = 1, 2, 3,…, n, i ≠ j)*, we assessed the similarity between the signal segments pair by means of calculating the Pearson’s correlation coefficient (Figure 2C). Taking the gyral vertex as an example, the correlation ${PCC}_{i,j}^{G}$ of signal segments between time window *i* and *j* was defined as:

$${PCC}_{i,j}^{G}=corrcoef\left(S_{i}^{G},S_{j}^{G}\right),i,j=1,2,3,...,n,i\neq j$$

We then defined the temporal variability of BOLD signal by means of calculating the variance of all correlation values between time window pairs (Figure 2D):

$$Var\left({PCC}^{G}\right)=\Sigma {\left({PCC}_{i,j}^{G}-\overset{¯}{{PCC}_{i,j}^{G}}\right)}^{2}/m,i,j=1,2,3,...,n,\text{i}\neq j,m=n(n-1)/2$$

where $\overset{¯}{{PCC}_{i,j}^{G}}$ is the mean correlation value. The potential difference of BOLD signal temporal variability among gyri, sulci, and undefined regions could then be assessed by means of comparing the mean temporal variance values of all vertices among the three groups. Since the fMRI signal represents the amplitude of BOLD oscillation, the correlation between different time windows of fMRI signals in Eq. (1) reflects the oscillation similarity of BOLD activity between different time windows. The Pearson’s correlation coefficient is not 0 across time, indicating that there is oscillation association of BOLD activity between different time windows. And the temporal variance of the oscillation similarity of BOLD activity is finally calculated among all time window pairs to assess the oscillation change of BOLD activity during the entire period of the time series in Eq. (2). A previous study (Zhang et al., 2016) has demonstrated that the variability of a brain region is modulated by its BOLD activity, the α band power of its EEG, and the ratio of intra- to inter-community structural connections. The temporal variability of BOLD signal is also suggested to learning performance (Bassett et al., 2011, 2013, 2015; Zhang et al., 2016) and intelligence (Saxe et al., 2018). Taken together, the abovementioned method is reasonable to assess the temporal variance of BOLD activity.

### Correlation of Gyral-Sulcal Temporal Variability With Fluid Intelligence Measures

To investigate the association between temporal variability of functional activity and the fluid intelligence, we performed correlation analysis of the variance value of each vertex with each of the three fluid intelligence measures. Specifically, since each vertex of the grayordinate data had correspondence across different subjects, we correlated the variance value of signal temporal variability with each of the three fluid intelligence measures for each vertex. Those vertices with significant correlation values were counted for gyri, sulci, and undefined regions, respectively, in order to assess the distribution of those vertices on gyri, sulci, and undefined regions. The potential difference of significantly correlated vertex distribution among gyri, sulci, and undefined regions could then be compared across subjects. Note that we performed above-mentioned comparisons in both whole-brain scale and region of interest (ROI)-scale. The whole-brain scale comparison helped the assessment of averaged gyri-sulci-undefined regions difference across all brain regions, while the ROI-scale comparison enabled the assessment of gyri-sulci-undefined regions difference within the same brain region. Based on previous findings (Duncan et al., 2000), we adopted four ROIs provided in the used HCP dataset which are related to fluid intelligence: Rostral Middle Frontal, SupraMarginal, Inferior Parietal, and Lateral Occipital regions. Visualization of the four selected ROIs is in Supplementary Figure 1.

## Results

### Temporal Variability Difference Between Gyri and Sulci

**FIGURE 3:**
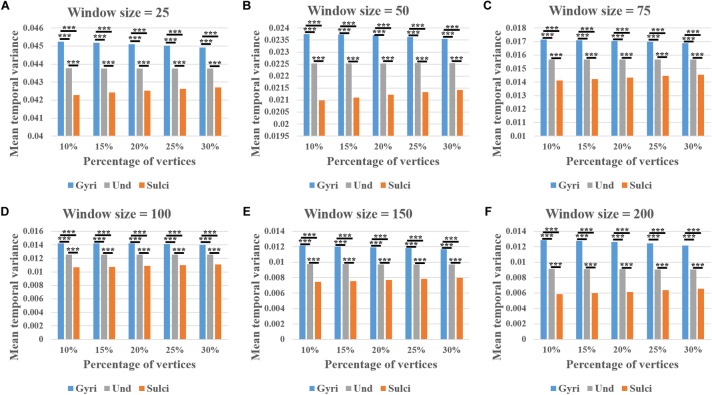
Mean temporal variance difference of functional activity among gyri, sulci, and undefined regions across all brain regions in a single subject. **(A–F)** The mean temporal variance of gyri, sulci, and undefined regions when window size equals 25, 50, 75, 100, 150, and 200, respectively. Within each sub-figure, five convexity threshold values (10, 15, 20, 25, and 30) corresponding to five percentages of vertices (10, 15, 20, 25, and 30%) are tested. ^∗∗∗^Indicates *p* < 0.001.

We found that the mean temporal variance of functional activity was significantly larger in gyri, smaller in sulci, and moderate in undefined regions across all brain regions within a single subject (independent sample *t*-test, *p* < 0.001, Bonferroni correction for multiple comparisons). Remarkably, this finding was consistent in thirty different combinations of six window size values (*l* = 25, 50, 75, 100, 150, and 200) and five convexity threshold values (*q* = 10, 15, 20, 25, and 30) shown in Figure 3A–F, respectively. More results of other subjects are provided in Supplementary Figures 2 – 5.

**FIGURE 4:**
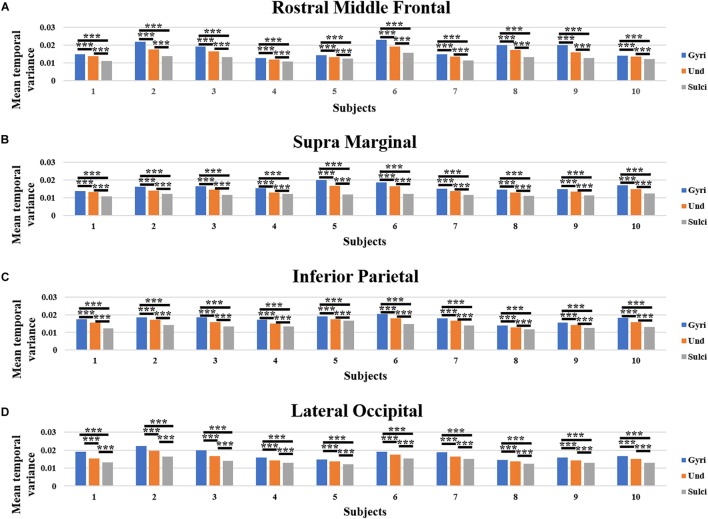
Mean temporal variance difference of functional activity among gyri, sulci, and undefined regions in ROI-scale in ten subjects. **(A–D)** The mean temporal variance of gyri, sulci, and undefined regions in ten subjects within each of the four ROIs, respectively. ^∗∗∗^Indicates *p* < 0.001.

Moreover, we obtained similar findings in ROI-scale comparison. Within each of the four ROIs, the mean temporal variance of functional activity was significantly larger in gyri, smaller in sulci, and moderate in undefined regions (independent sample *t*-test, *p* < 0.001, Bonferroni correction for multiple comparisons). Figure 4A–D presents the mean temporal variance among gyri, sulci, and undefined regions in ten subjects within each of the four ROIs, respectively.

### Reproducibility of Temporal Variability Difference via Permutation Test

We further assessed the reproducibility of temporal variability difference among gyral/sulcal/undefined vertices via permutation test. For each subject, we randomly assigned all vertices into 10% gyri, 10% sulci, and 80% undefined groups in line with the convexity threshold value 10, and compared the mean temporal variance of functional activity among the three groups. The procedure was repeated for 1000 times. We found that the mean temporal variance of functional activity was still significantly larger in gyri, smaller in sulci, and moderate in undefined regions (1000-time permutation independent sample *t*-test, *p* < 0.05, Bonferroni correction for multiple comparisons).

### Correlation Between Temporal Variability and Fluid Intelligence Measures

**FIGURE 5:**
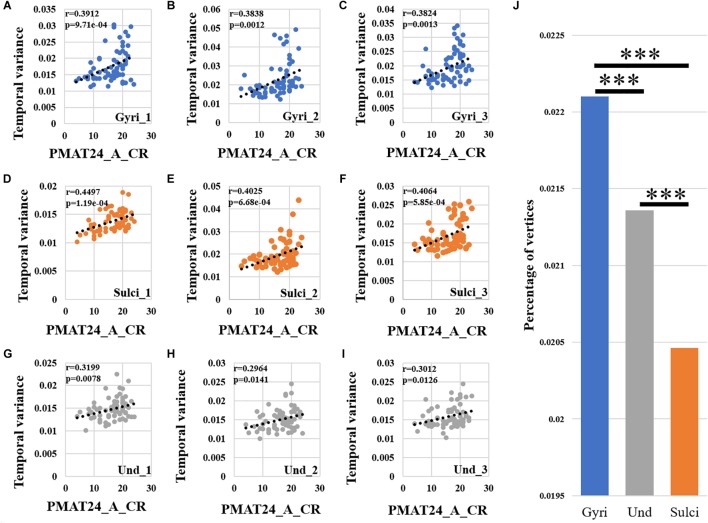
Correlation between temporal variability of functional activity and fluid intelligence measure “number of correct responses” (PMAT24_A_CR) across all brain regions. **(A–C)** The correlations between temporal variance and “number of correct responses” measure across all 68 subjects of three example gyral vertices, respectively. **(D–F)** The correlations between temporal variance and “number of correct responses” measure across all 68 subjects of three example sulcal vertices, respectively. **(G–I)** The correlations between temporal variance and “number of correct responses” measure across all 68 subjects of three example undefined vertices, respectively. **(J)** The mean percentage of gyral/sulcal/undefined vertices with significant positive correlations with the “number of correct responses” measure across all 68 subjects. ^∗∗∗^Indicates *p* < 0.001.

**FIGURE 6:**
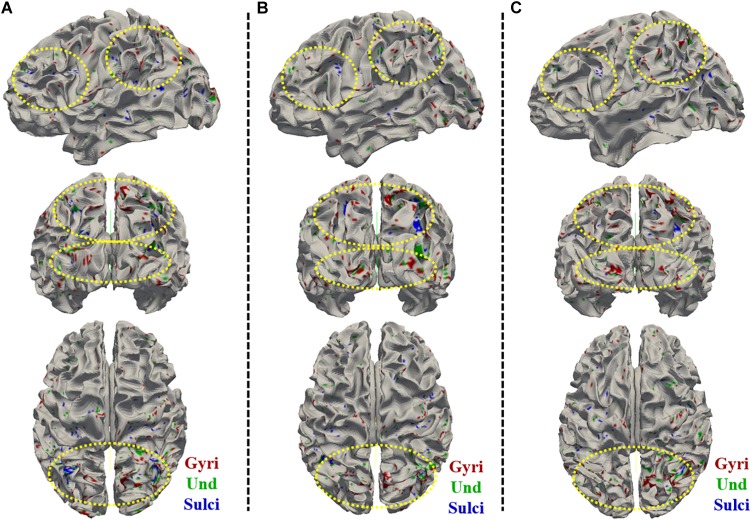
Visualization of the vertices with significant positive correlation between temporal variance and the “number of correct responses” measure across all brain regions on cortical surface across three subjects. **(A–C)** shows the vertices distribution on cortical surface from three views across three subjects, respectively. The main locations of those vertices on cortical surface are highlighted by yellow dashed ovals.

We found that the temporal variability of functional activity in certain gyral/sulcal/undefined vertices had significant positive correlation with each of the three fluid intelligence measures (*r* > 0.2, *p* < 0.05, Bonferroni correction for multiple comparisons) across all brain regions. Taking the “number of correct responses” measure as an example, Figure 5A–C present the correlations between temporal variance and this measure across all 68 subjects of three example gyral vertices, three example sulcal vertices in Figure 5D–F, and three undefined vertices in Figure 5G–I. Moreover, those vertices which are significantly positively correlated with the “number of correct responses” measure are visualized on the cortical surface shown in Figure 6 and are mainly located in the middle frontal cortex, inferior parietal lobe and visual cortex. More results of the other two fluid intelligence measures are provided in Supplementary Figures 6–9.

Moreover, we found that for those vertices with significant positive correlations between temporal variance and the “number of correct responses” measure across all brain regions, the percentage of vertices was significantly larger in gyri, smaller in sulci, and moderate in undefined regions across all 68 subjects as presented in Figure 5J (independent sample *t*-test, *p* < 0.001, Bonferroni correction for multiple comparisons). For the other two measures “median reaction time for correct responses” and “total skipped items,” the percentage of vertices was significantly smaller in gyri, larger in sulci, and moderate in undefined regions across all 68 subjects as presented in Supplementary Figures 6J, 7J (independent sample *t*-test, *p* < 0.001, Bonferroni correction for multiple comparisons).

**FIGURE 7:**
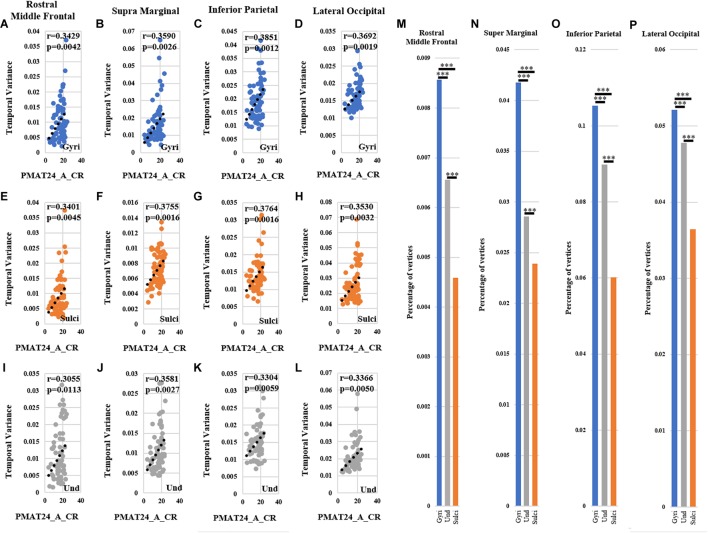
Correlation between temporal variability of functional activity and fluid intelligence measure “number of correct responses” (PMAT24_A_CR) within each of the four ROIs. **(A–D)** The correlations between temporal variance and “number of correct responses” measure across all 68 subjects in one example gyral vertex within each of the four ROIs, respectively. **(E–H)** The correlations between temporal variance and “number of correct responses” measure across all 68 subjects in one example sulcal vertex within each of the four ROIs, respectively. **(I–L)** The correlations between temporal variance and “number of correct responses” measure across all 68 subjects in one example undefined vertex within each of the four ROIs, respectively. **(M–P)** The mean percentage of gyral/sulcal/undefined vertices with significant positive correlations with the “number of correct responses” measure across all 68 subjects. ^∗∗∗^Indicates *p* < 0.001.

**FIGURE 8:**
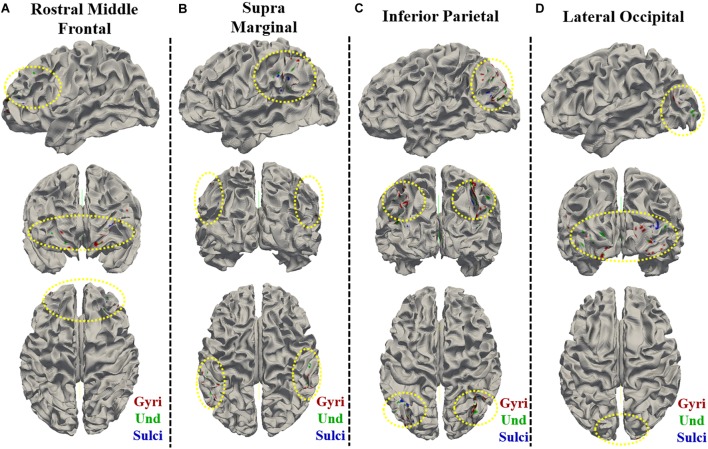
Visualization of the vertices with significant positive correlation between temporal variance and the “number of correct responses” measure within single brain region on cortical surface of one subject. The vertices distribution on cortical surface are shown from three views within **(A)** Rostral Middle Frontal, **(B)** SupraMarginal, **(C)** Inferior Parietal, and **(D)** Lateral Occipital regions, respectively. The main locations of those vertices on cortical surface are highlighted by yellow dashed ovals.

For ROI-scale comparison, we obtained similar findings as the whole-brain comparison. Within each of the four ROIs, the temporal variability of functional activity in certain gyral/sulcal/undefined vertices had significant positive correlation with each of the three fluid intelligence measures (*r* > 0.2, *p* < 0.05, Bonferroni correction for multiple comparisons) as illustrated in Figure 7A–L and Supplementary Figures 10, 11. Those vertices which are significantly positively correlated with the three fluid intelligence measures within each of the four regions are visualized on the cortical surface shown in Figure 8 and Supplementary Figures 12, 13. Moreover, for those vertices with significant positive correlations between temporal variance and the “number of correct responses” measure within each of the four regions, the percentage of vertices was significantly larger in gyri, smaller in sulci, and moderate in undefined regions across all 68 subjects as presented in Figure 7M–P (independent sample *t*-test, *p* < 0.001, Bonferroni correction for multiple comparisons). For the other two measures “median reaction time for correct responses” and “total skipped items,” the percentage of vertices was significantly smaller in gyri, larger in sulci, and moderate in undefined regions in three regions while no significant difference in middle frontal cortex as presented in Supplementary Figures 10, 11 (independent sample *t*-test, *p* < 0.001, Bonferroni correction for multiple comparisons).

Furthermore, we adopted all 12 temporal variance values of gyri/sulci/undefined regions in the four ROIs as independent variables to predict the fluid intelligence measure “number of correct responses.” Specifically, we adopted linear regression model and leave-one-out cross validation implemented in Weka software. The result showed that the correlation coefficient value indicating how well the predictions are correlated with the actual value is as high as 0.41, suggesting that the temporal variance values of gyri/sulci/undefined regions in the four ROIs can predict the fluid intelligence.

## Discussion

We found that the temporal variance of resting state fMRI BOLD signal was significantly larger in gyri, smaller in sulci, and moderate in undefined regions. Since the undefined regions were intermixed areas between gyri crown and sulci fundi, it was reasonable that their associated temporal variance of resting state fMRI BOLD signal was moderate between gyri and sulci. Notably, the current findings were consistent across different subjects, with a variety of combinations of time window size and convexity threshold values, and under different vertices selection strategies (convexity threshold and permutation test), demonstrating that gyri truly had significant larger temporal variance of resting state functional activity compared with sulci. This finding was supported by our recent study (Liu et al., 2018) showing that gyral resting state fMRI BOLD signals had lower frequency than sulcal signals. The fMRI BOLD signals with lower frequency tend to have more global temporal changes. Therefore, gyral resting state BOLD signals were more variable in global temporal changes represented as temporal variance than sulcal signals in this study.

We found that the temporal variability of functional activity in the middle frontal cortex, inferior parietal lobe, and visual cortex had significant positive correlation with the fluid intelligence measures. Previous studies have argued that fluid intelligence was linked to variations in human neuronal structure and function (Duncan et al., 2000; Duncan, 2003, 2005). The brain with higher variability of functional activity might be more easily for learning and higher-order relational reasoning of variable external environments (Bassett et al., 2011, 2013, 2015; Zhang et al., 2016; Saxe et al., 2018). Therefore, the identified positive correlation between temporal variability of functional activity and fluid intelligence measures was reasonable. The current finding was also consistent with previous studies reporting the correlation between fluid intelligence related networks and individual fluid intelligence scores (Santarnecchi et al., 2017), and that brain entropy measured by resting state fMRI was positively associated with intelligence (Saxe et al., 2018). Moreover, it was reported that in a spatial task with high Spearman’s *g* (general intelligence) involvement, there were high-*g* activations occurred bilaterally in the prefrontal cortex, parietal lobe, and visual cortex (Duncan et al., 2000). The prefrontal cortex was recruited by different forms of cognitive demand such as working memory load, task novelty, response competition, and perceptual difficulty, etc. (Duncan and Owen, 2000) which were critical building blocks for fluid intelligence. The parietal lobe cortex was recruited in a variety of visuospatial task (Corbetta et al., 1995). The recruited visual cortex might presumably reflect more extensive visual analysis and/or the effects of eye movements (Duncan et al., 2000).

We found that the vertices with significant positive correlations between temporal variability and the fluid intelligence measure “number of correct responses” were predominately located in gyri, moderate in undefined regions, and less in sulci, while the other two measures “median reaction time for correct responses” and “total skipped items” generally showed reversed distributions, i.e., more in sulci, moderate in undefined regions, and less in gyri (Supplementary Figures 6, 7, 10, 11). This is reasonable since higher fluid intelligence corresponded to more “number of correct responses,” and less “median reaction time for correct responses” and “total skipped items.” As visualized in Supplementary Figures 6, 7, 10, 11, a number of data points may qualify as outliers and thus potentially bias the correlation. Moreover, in Supplementary Figures 7, 11 there is a population of “no skipped item” subjects (26 out of 68 subjects) whose temporal variance spans almost the entire range of temporal variations in the sample, which may weaken the correlation. It is suggested that the “median reaction time for correct responses” and “total skipped items” measure of fluid intelligence may not have as strong correlations with temporal variations as the “number of correct responses” (Figure 5). Nevertheless, the positive correlation between the temporal variations and the first fluid intelligence measure “number of correct responses” is significant and supports our conclusion. This finding provided more evidence to elucidate the temporal variability difference of functional activity among gyri, sulci, and undefined regions. That is, gyri might participate more in the fluid intelligence than sulci and undefined regions. Our previous studies have demonstrated that gyri represent a global functional hub that performs neural communication among remote brain regions, and sulci a local function processing unit that directly communicates with neighboring gyri (Deng et al., 2014). The fluid intelligence might be more related to and supported by global neural communication among gyral regions in the whole brain.

This study assessed the temporal variability of functional activity using resting state fMRI signals in line with previous study (Zhang et al., 2016), since the resting state fMRI signals represent intrinsic functional brain activity without being affected by any external stimuli in task-based fMRI. Illustration of the temporal variability mapped on cortex with different time windows is in Supplementary Figure 14. Therefore, it would be more suitable in the present study to assess the correlation between temporal variability of intrinsic functional activity and general human intelligence which is not related to specific task stimulus. In the future, it would be interesting to investigate the relationship between temporal variability of task-based fMRI signals and the in-task performance under specific task stimuli.

The present study has several limitations. First, the current study used average convexity (see section “Cortical Surface Parcellation of Gyri and Sulci”) to define gyri/sulci within the brain regions. However, certain brain regions, e.g., insula, are hard to be defined as gyri/sulci merely based on the average convexity information. Although those brain regions were not identified to be related to fluid intelligence in the current study, more cautiousness is needed to study the gyral/sulcal characteristics of those regions in the future. Second, the current study adopted HCP grayordinate rsfMRI data to explore the temporal variability of BOLD signals on gyri/sulci. Due to the natural characteristics of fMRI imaging, more direct evidence in a finer-scale (e.g., neuron-level) is needed in the future to further validate the current findings of temporal variability difference between gyri and sulci.

In conclusion, the present study is one of the first studies to assess the temporal variability characteristics of resting state functional activity in gyri and sulci as well as its association with behavioral measures (fluid intelligence in this study). The findings could provide novel insights to understand the functional mechanisms of gyri and sulci and to demonstrate their functional relevance on the behavioral level. A future work would be applying the analysis framework on more subjects and to correlate with other behavioral measures which might be scientifically related to temporal variability. It would be also interesting to compare the temporal variability of functional activity between resting state and task-based fMRI signals in gyri and sulci and its association with behavioral measures.

## Ethics Statement

This study used publicly released Human Connectome Project dataset.

## Author Contributions

SY analyzed part of the data, generated the figures, and wrote the manuscript draft. ZZ, HC, LZ, ZH, and HL analyzed part of the data. TZ interpreted part of the results and guided LZ and ZH on this study. LG guided LZ, ZH, and HL on this study. TL interpreted part of the results and revised the manuscript. BB and KK interpreted the results and revised the manuscript. XJ designed the study, interpreted the results, and critically revised the figures and manuscript.

## Conflict of Interest Statement

The authors declare that the research was conducted in the absence of any commercial or financial relationships that could be construed as a potential conflict of interest.
