# Supplementary material for: Temporal Variability of Cortical Gyral-Sulcal Resting State Functional Activity Correlates With Fluid Intelligence
Source: Front Neural Circuits. 2019 May 15;13:36. doi: 10.3389/fncir.2019.00036 (PMC6529596; doi:10.3389/fncir.2019.00036)
Supplement: Supplementary file 1 [file Data_Sheet_1.docx]

Supplementary Material

Temporal Variability of Cortical Gyral-Sulcal Resting State Functional Activity Correlates with Fluid Intelligence

Shimin Yang^1^, Zhongbo Zhao^1^, Han Cui^1^, Tuo Zhang^2^, Lin Zhao^2^, Zhibin He^2^, Huan Liu^2^, Lei Guo^2^, Tianming Liu^3^, Benjamin Becker^1^, Keith M Kendrick^1^, Xi Jiang^1*^

^1^The Clinical Hospital of Chengdu Brain Science Institute, MOE Key Lab for Neuroinformation, School of Life Science and Technology, University of Electronic Science and Technology of China, Chengdu, China

^2^School of Automation, Northwestern Polytechnical University, Xi’an, China

^3^Department of Computer Science and Bioimaging Research Center, The University of Georgia, Athens, GA, USA

*** Correspondence:** Xi Jiang: xijiang@uestc.edu.cn

# Supplementary Figures and Tables

## Supplementary Figures


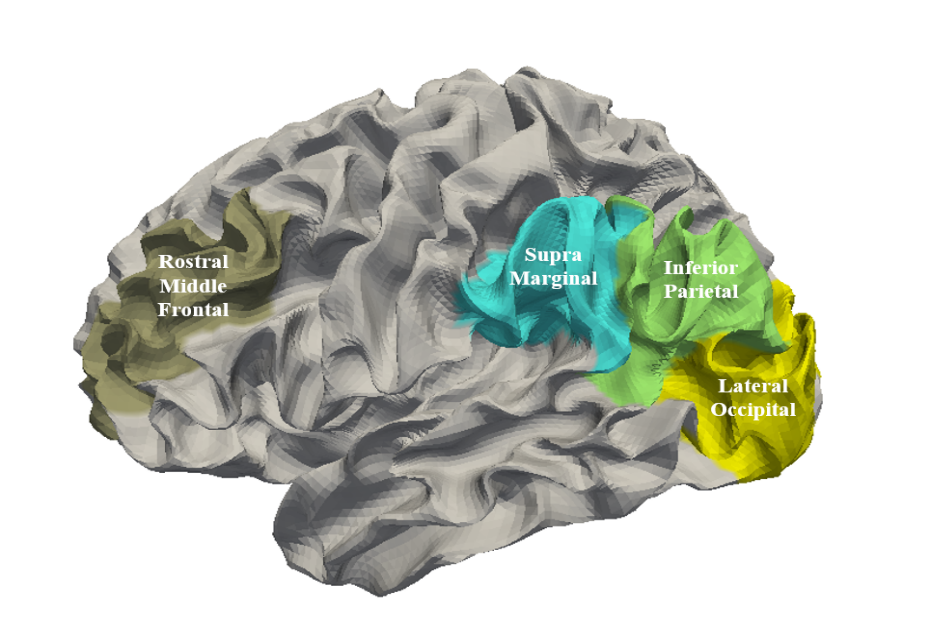


**Supplementary Figure 1.** Visualization of the four selected regions of interest (ROIs) for gyri-sulci-undefined comparison in ROI-scale. The left hemisphere is shown as an example.


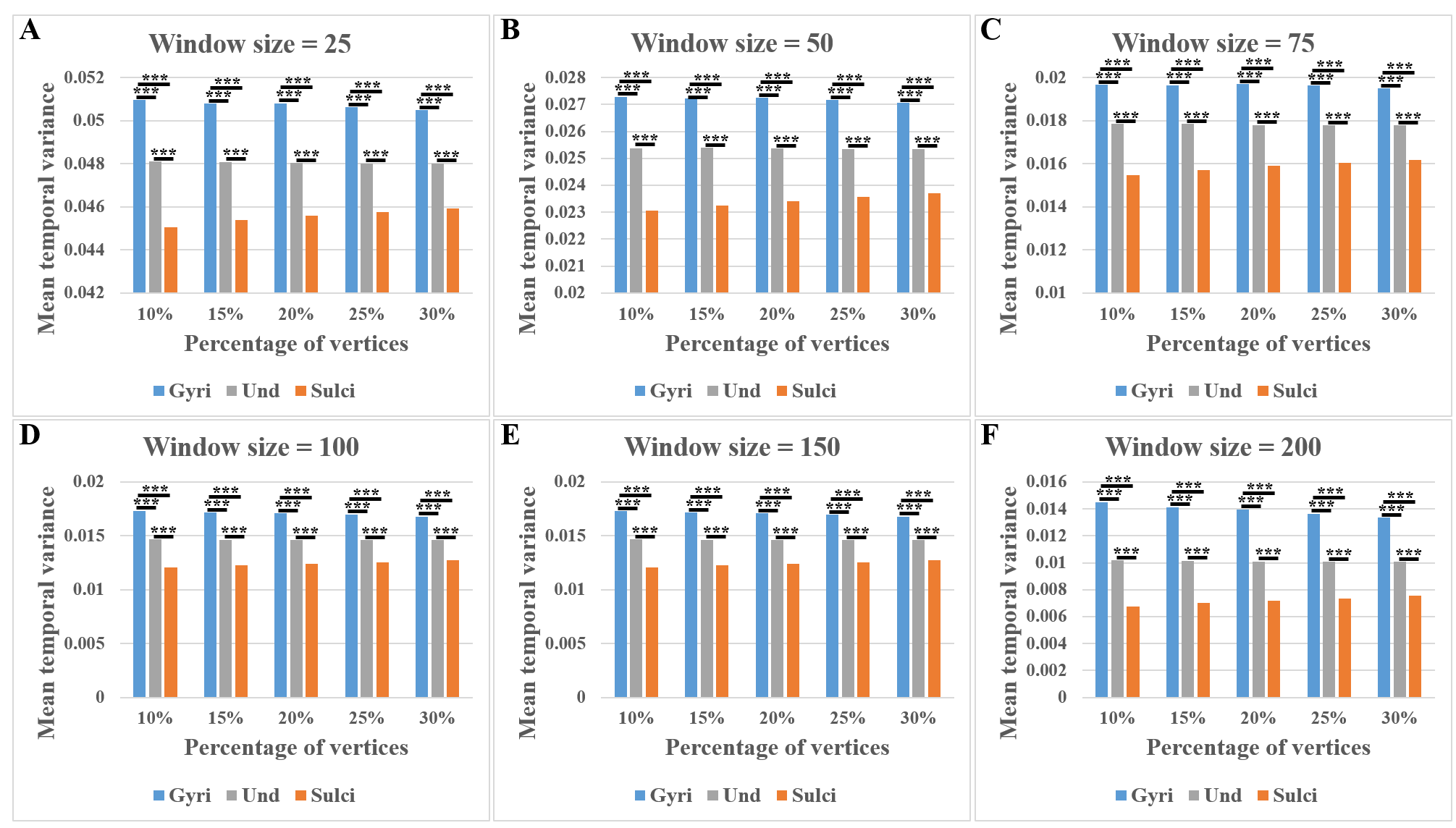


**Supplementary Figure 2.** Mean temporal variance difference of functional activity among gyri, sulci, and undefined regions across all brain regions in a single subject. (A)-(F): the mean temporal variance of gyri, sulci, and undefined regions when window size equals 25, 50, 75, 100, 150, and 200, respectively. Within each sub-figure, five convexity threshold values (10, 15, 20, 25, and 30) corresponding to five percentages of vertices (10%, 15%, 20%, 25%, and 30%) are tested. *** indicates *p* < 0.001.


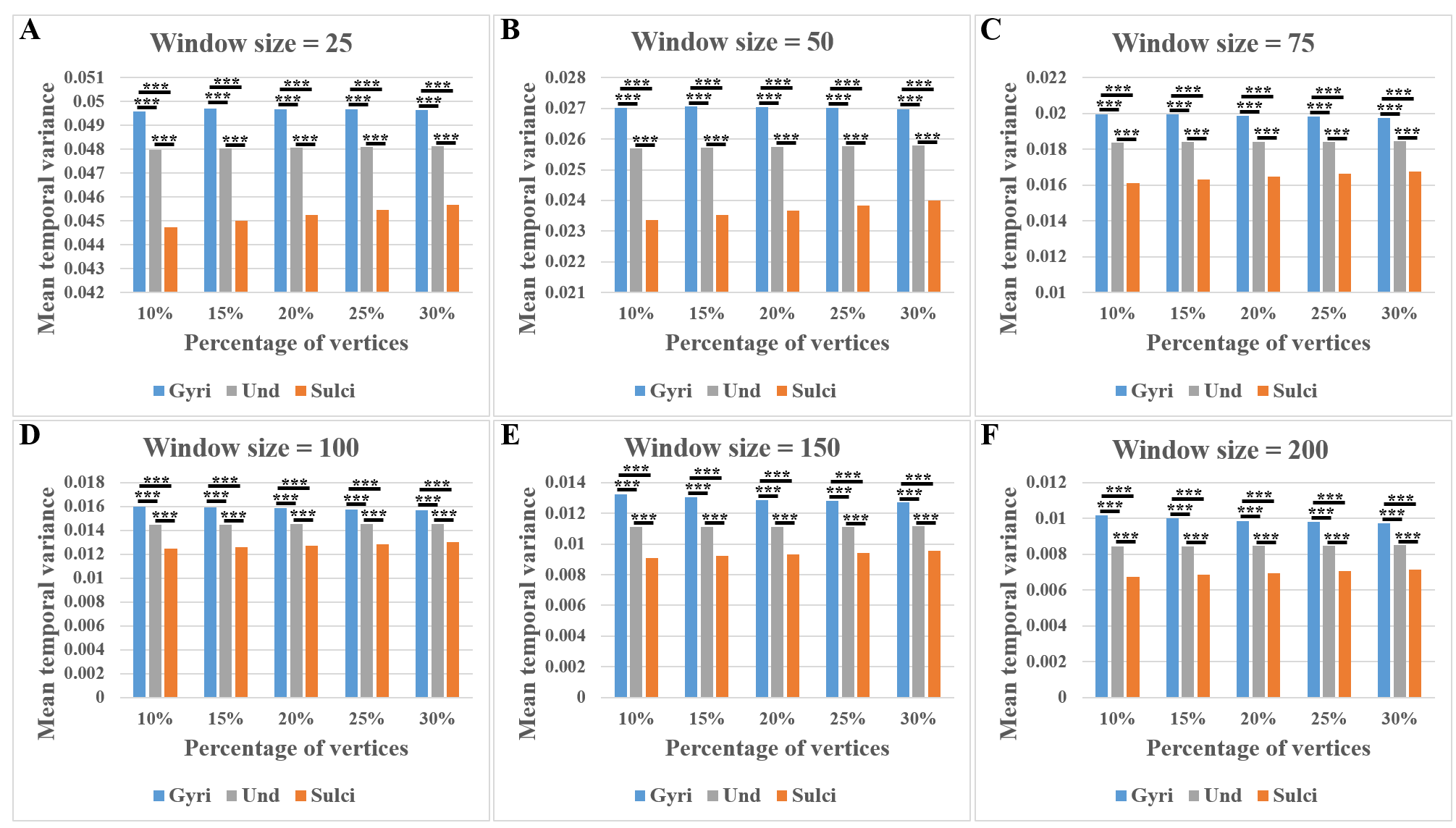


**Supplementary Figure 3.** Mean temporal variance difference of functional activity among gyri, sulci, and undefined regions across all brain regions in a single subject. (A)-(F): the mean temporal variance of gyri, sulci, and undefined regions when window size equals 25, 50, 75, 100, 150, and 200, respectively. Within each sub-figure, five convexity threshold values (10, 15, 20, 25, and 30) corresponding to five percentages of vertices (10%, 15%, 20%, 25%, and 30%) are tested. *** indicates *p* < 0.001.


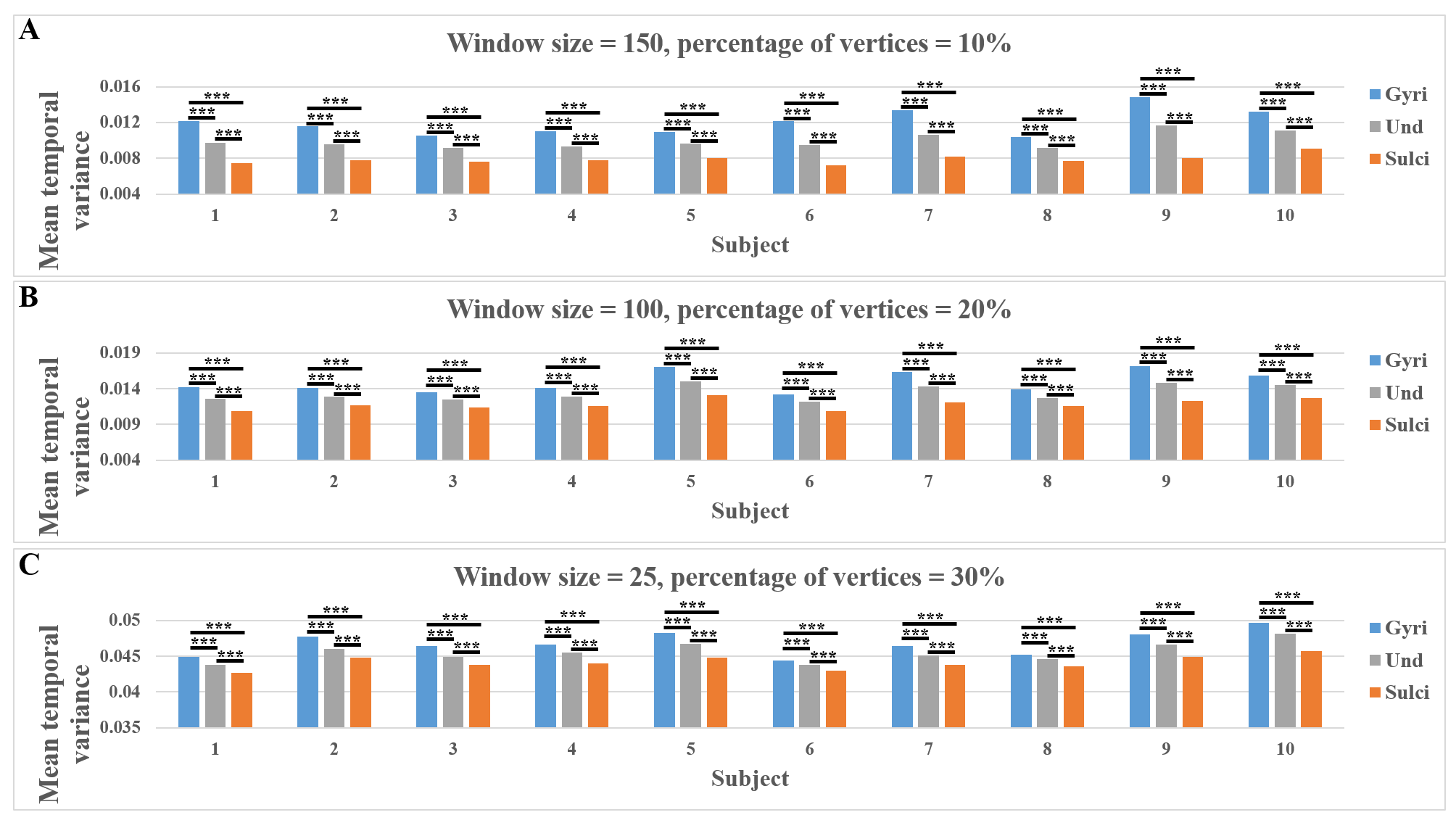


**Supplementary Figure 4.** Mean temporal variance difference of functional activity among gyri, sulci, and undefined regions across all brain regions in ten subjects. (A)-(C): The mean temporal variance of gyri, sulci, and undefined regions across ten subjects with three example combinations of window size and convexity threshold value, respectively. *** indicates *p* < 0.001.


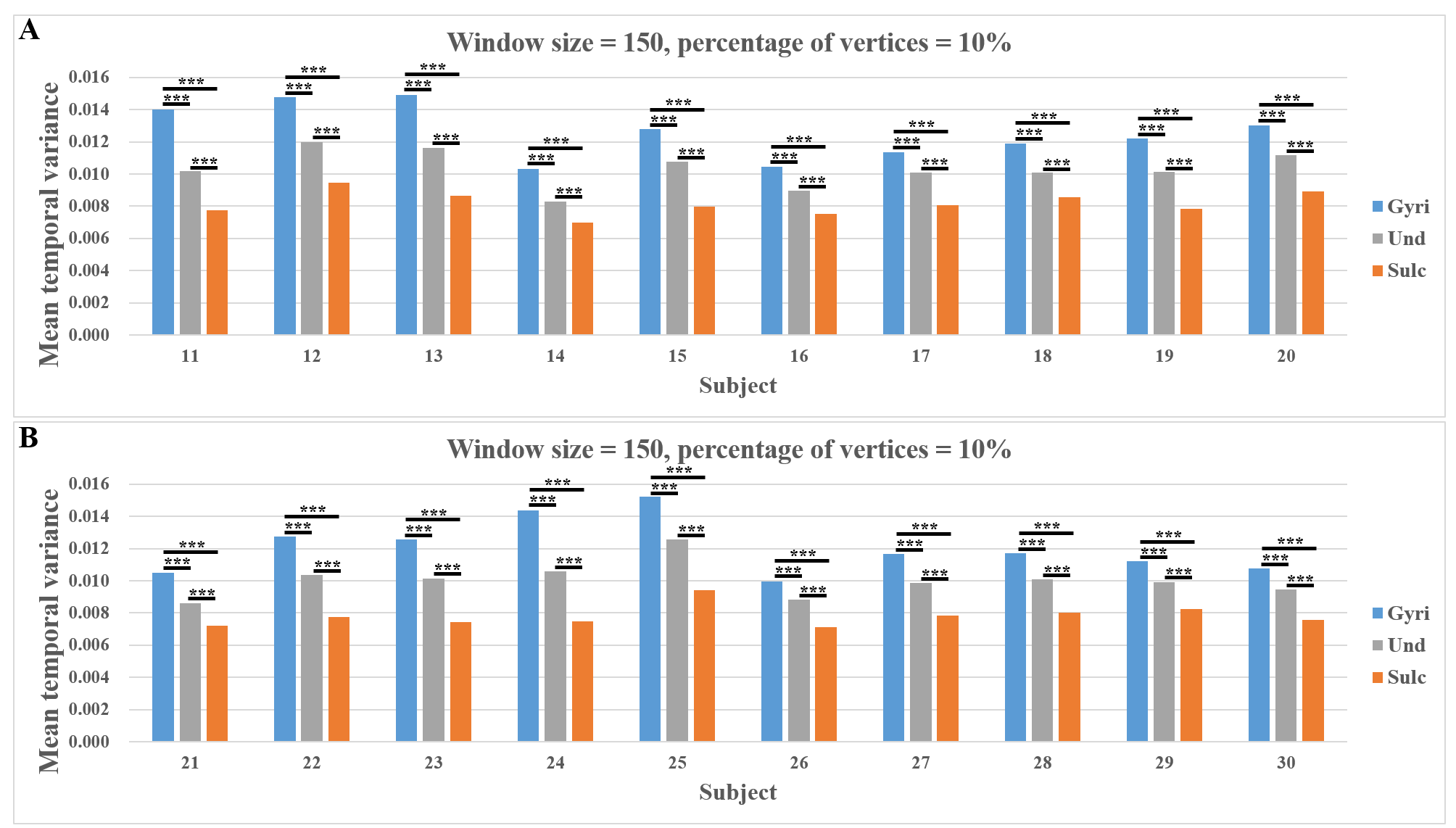


**Supplementary Figure 5.** Mean temporal variance difference of functional activity among gyri, sulci, and undefined regions across all brain regions in another twenty subjects. (A)-(B): The mean temporal variance of gyri, sulci, and undefined regions with window size 150 and convexity threshold value 10 across ten subjects, respectively. *** indicates *p* < 0.001.


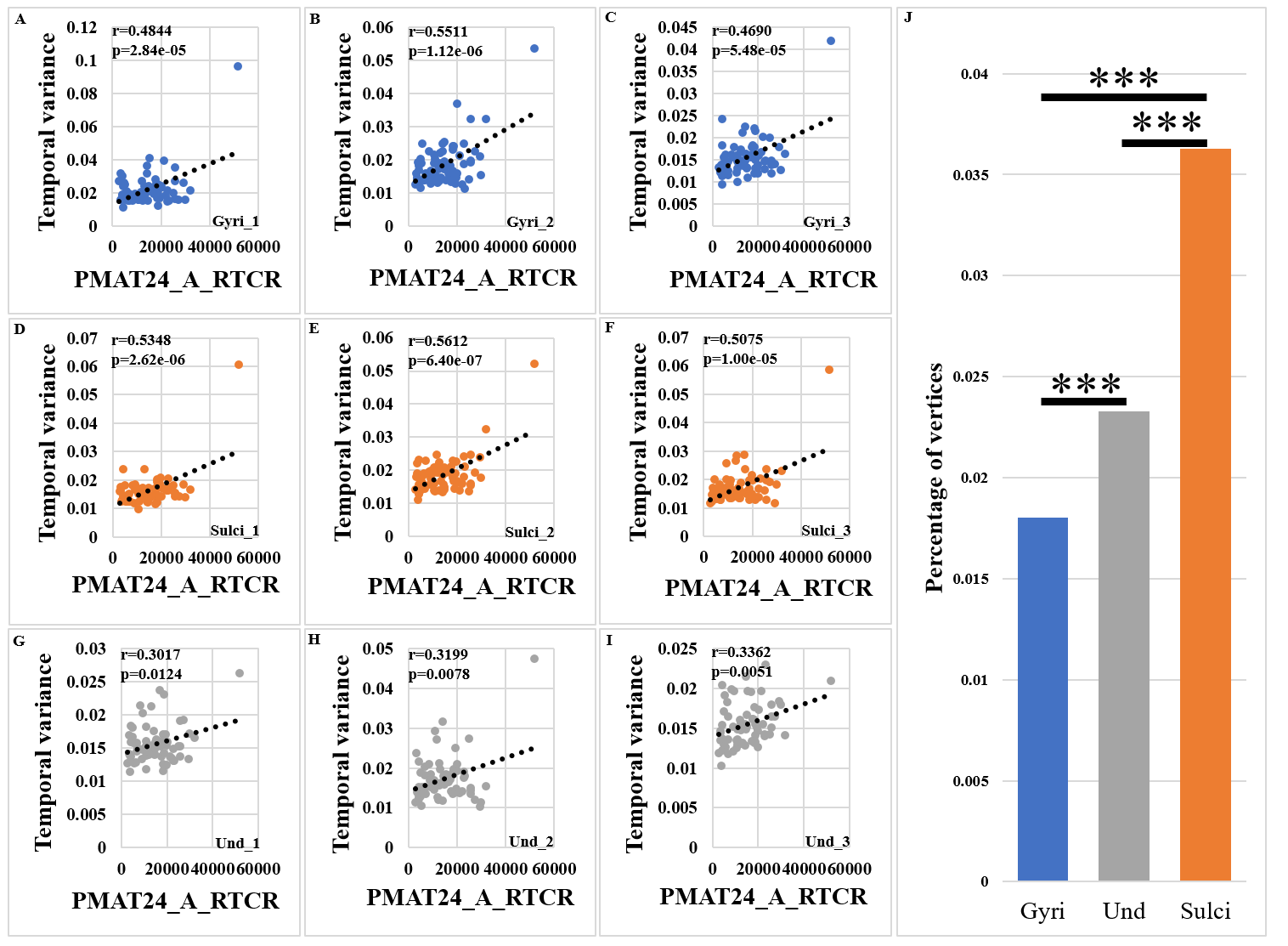


**Supplementary Figure 6.** Correlation between temporal variability of functional activity and fluid intelligence measure ‘median reaction time for correct responses’ (PMAT24_A_RTCR) across all brain regions. (A)-(C): The correlations between temporal variance and ‘median reaction time for correct responses’ measure across all 68 subjects of three example gyral vertices, respectively. (D)-(F): The correlations between temporal variance and ‘median reaction time for correct responses’ measure across all 68 subjects of three example sulcal vertices, respectively. (G)-(I): The correlations between temporal variance and ‘median reaction time for correct responses’ measure across all 68 subjects of three example undefined vertices, respectively. (J): The mean percentage of gyral/sulcal/undefined vertices with significant positive correlations with the ‘median reaction time for correct responses’ measure across all 68 subjects. *** indicates *p* < 0.001.


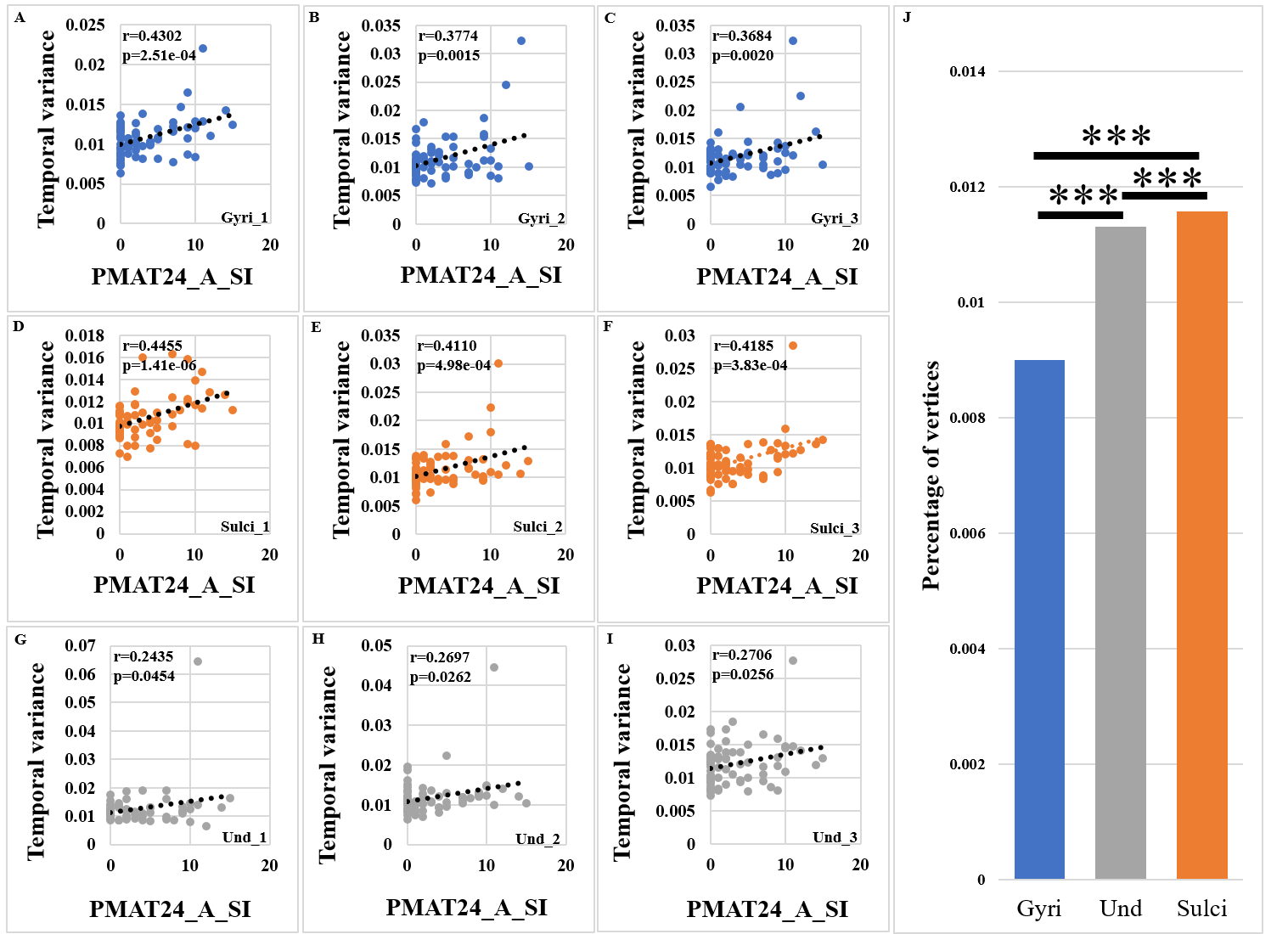


**Supplementary Figure 7.** Correlation between temporal variability of functional activity and fluid intelligence measure ‘total skipped items’ (PMAT24_A_SI) across all brain regions. (A)-(C): The correlations between temporal variance and ‘total skipped items’ measure across all 68 subjects of three example gyral vertices, respectively. (D)-(F): The correlations between temporal variance and ‘total skipped items’ measure across all 68 subjects of three example sulcal vertices, respectively. (G)-(I): The correlations between temporal variance and ‘total skipped items’ measure across all 68 subjects of three example undefined vertices, respectively. (J): The mean percentage of gyral/sulcal/undefined vertices with significant positive correlations with the ‘total skipped items’ measure across all 68 subjects. *** indicates *p* < 0.001.


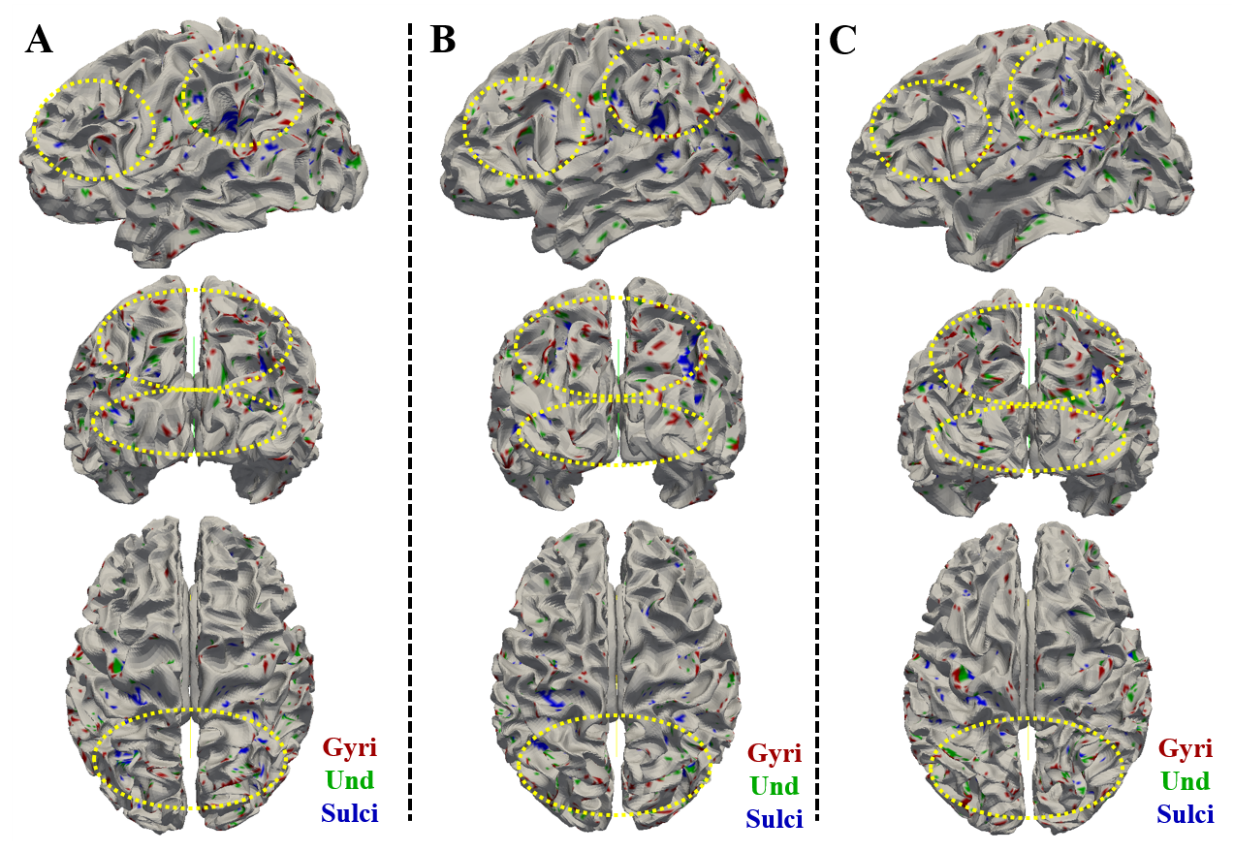


**Supplementary Figure 8.** Visualization of the vertices with significant positive correlation between temporal variance and the ‘median reaction time for correct responses’ measure across all brain regions on cortical surface across three subjects. (A)-(C) shows the vertices distribution on cortical surface from three views across three subjects, respectively. The main locations of those vertices on cortical surface are highlighted by yellow dashed ovals.


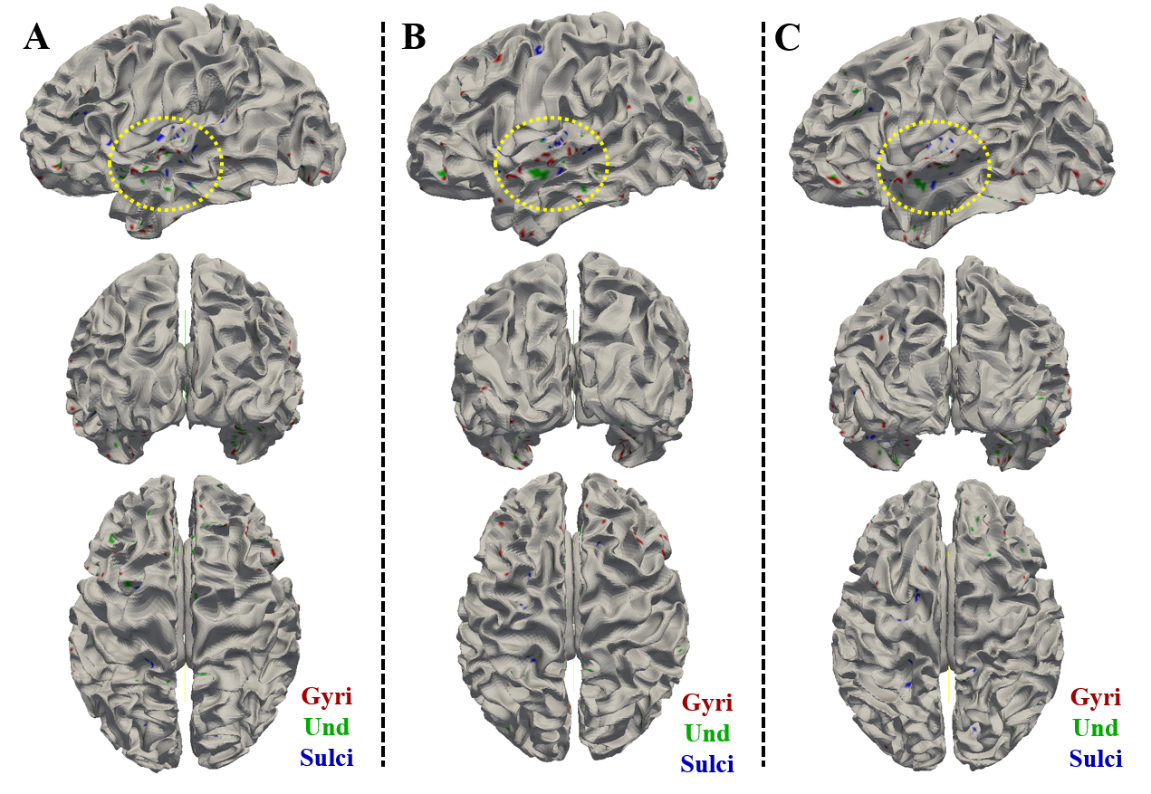


**Supplementary Figure 9.** Visualization of the vertices with significant positive correlation between temporal variance and the ‘total skipped items’ measure across all brain regions on cortical surface across three subjects. (A)-(C) shows the vertices distribution on cortical surface from three views across three subjects, respectively. The main locations of those vertices on cortical surface are highlighted by yellow dashed ovals.


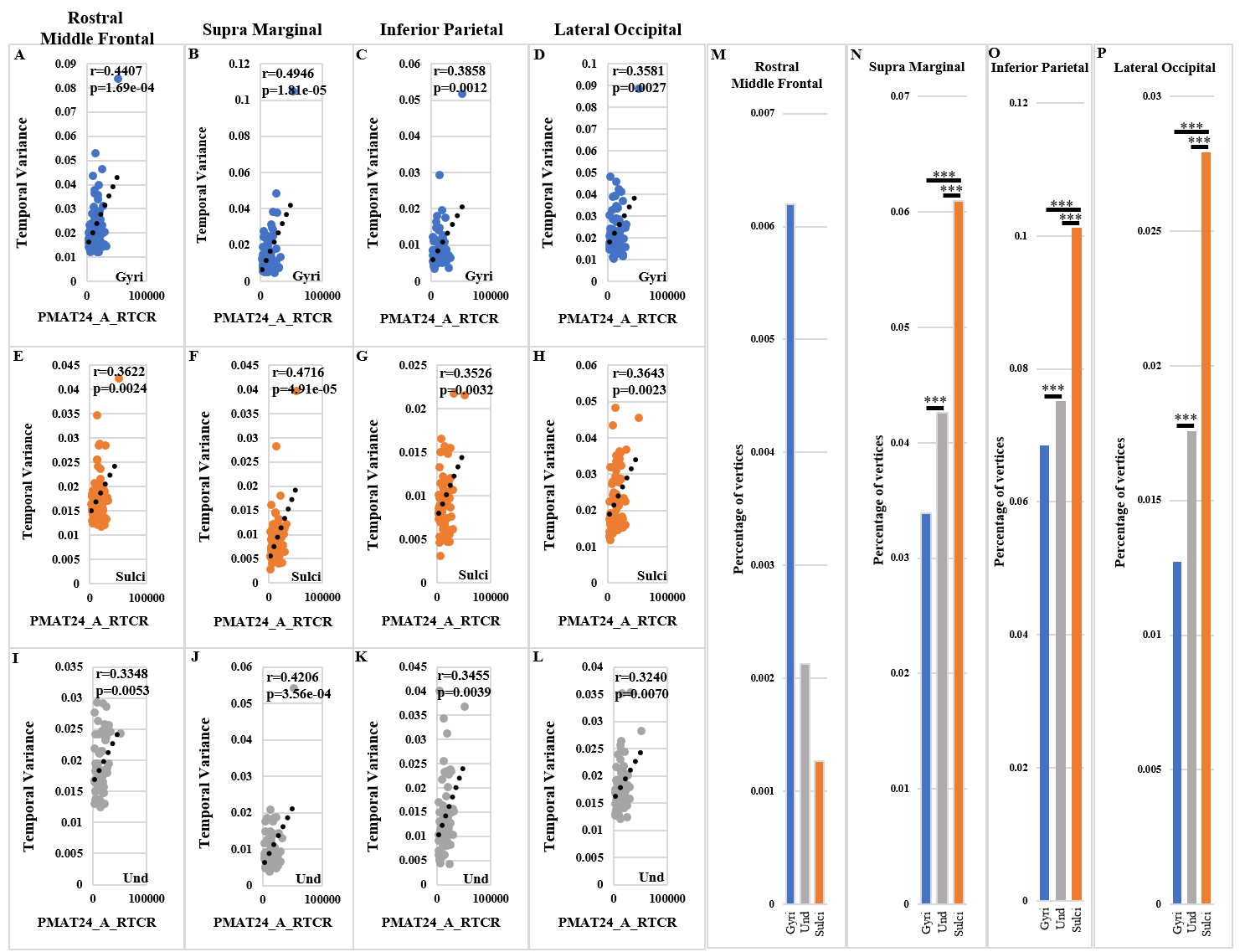


**Supplementary Figure 10.** Correlation between temporal variability of functional activity and fluid intelligence measure ‘median reaction time for correct responses’ (PMAT24_A_RTCR) within each of the four ROIs. (A)-(D): The correlations between temporal variance and ‘median reaction time for correct responses’ measure across all 68 subjects in one example gyral vertex within each of the four ROIs, respectively. (E)-(H): The correlations between temporal variance and ‘median reaction time for correct responses’ measure across all 68 subjects in one example sulcal vertex within each of the four ROIs, respectively. (I)-(L): The correlations between temporal variance and ‘median reaction time for correct responses’ measure across all 68 subjects in one example undefined vertex within each of the four ROIs, respectively. (M)-(P): The mean percentage of gyral/sulcal/undefined vertices with significant positive correlations with the ‘median reaction time for correct responses’ measure across all 68 subjects. *** indicates *p* < 0.001.


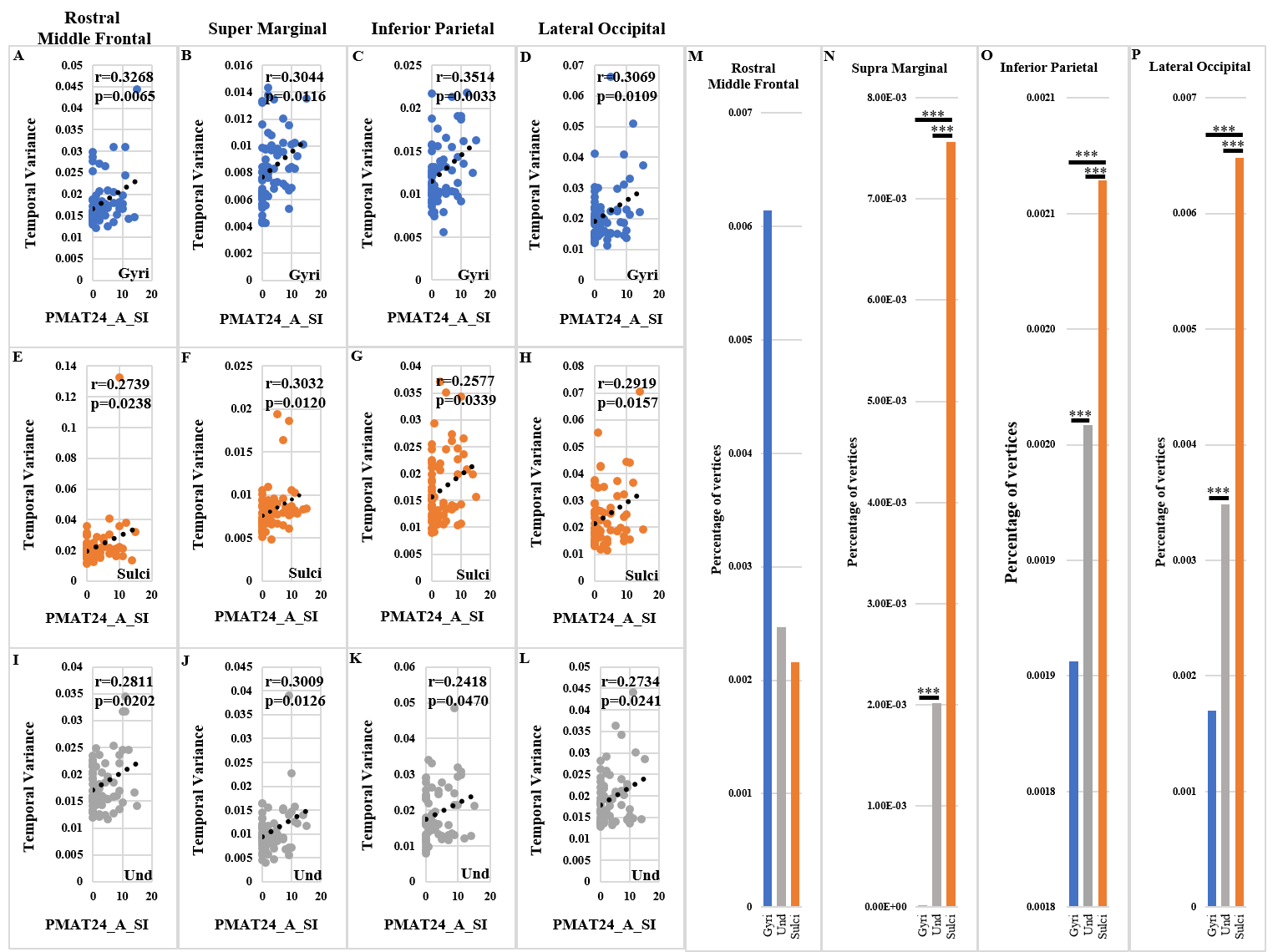


**Supplementary Figure 11.** Correlation between temporal variability of functional activity and fluid intelligence measure ‘total skipped items’ (PMAT24_A_SI) within each of the four ROIs. (A)-(D): The correlations between temporal variance and ‘total skipped items’ measure across all 68 subjects in one example gyral vertex within each of the four ROIs, respectively. (E)-(H): The correlations between temporal variance and ‘total skipped items’ measure across all 68 subjects in one example sulcal vertex within each of the four ROIs, respectively. (I)-(L): The correlations between temporal variance and ‘total skipped items’ measure across all 68 subjects in one example undefined vertex within each of the four ROIs, respectively. (M)-(P): The mean percentage of gyral/sulcal/undefined vertices with significant positive correlations with the ‘total skipped items’ measure across all 68 subjects. *** indicates *p* < 0.001.


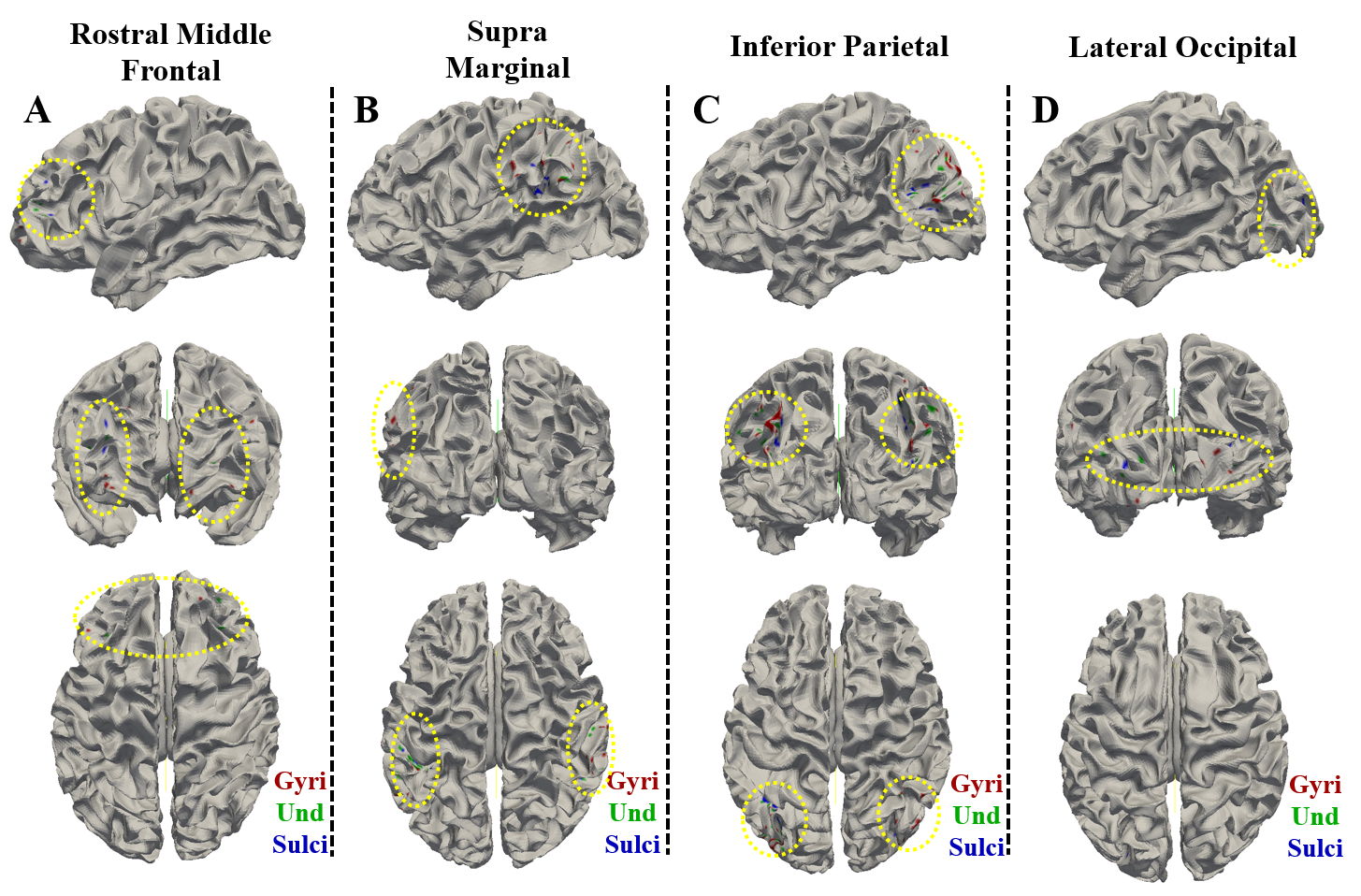


**Supplementary Figure 12.** Visualization of the vertices with significant positive correlation between temporal variance and the ‘median reaction time for correct responses’ measure within an ROI on cortical surface of one subject. The vertices distribution on cortical surface are shown from three views within (A) Rostral Middle Frontal, (B) SupraMarginal, (C) Inferior Parietal, and (D) Lateral Occipital regions, respectively. The main locations of those vertices on cortical surface are highlighted by yellow dashed ovals.


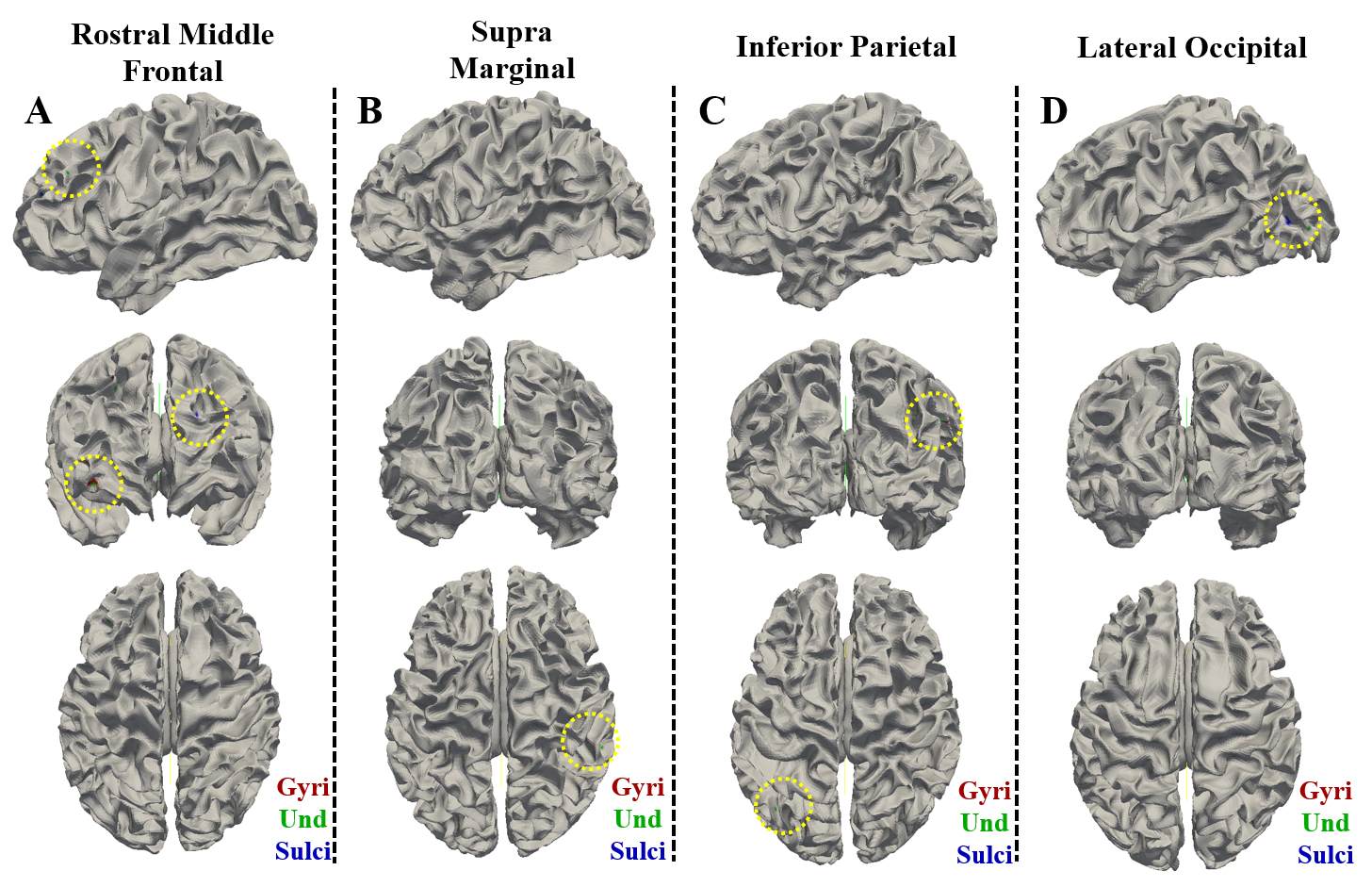


**Supplementary Figure 13.** Visualization of the vertices with significant positive correlation between temporal variance and the ‘total skipped items’ measure within an ROI on cortical surface of one subject. The vertices distribution on cortical surface are shown from three views within (A) Rostral Middle Frontal, (B) SupraMarginal, (C) Inferior Parietal, and (D) Lateral Occipital regions, respectively. The main locations of those vertices on cortical surface are highlighted by yellow dashed ovals.


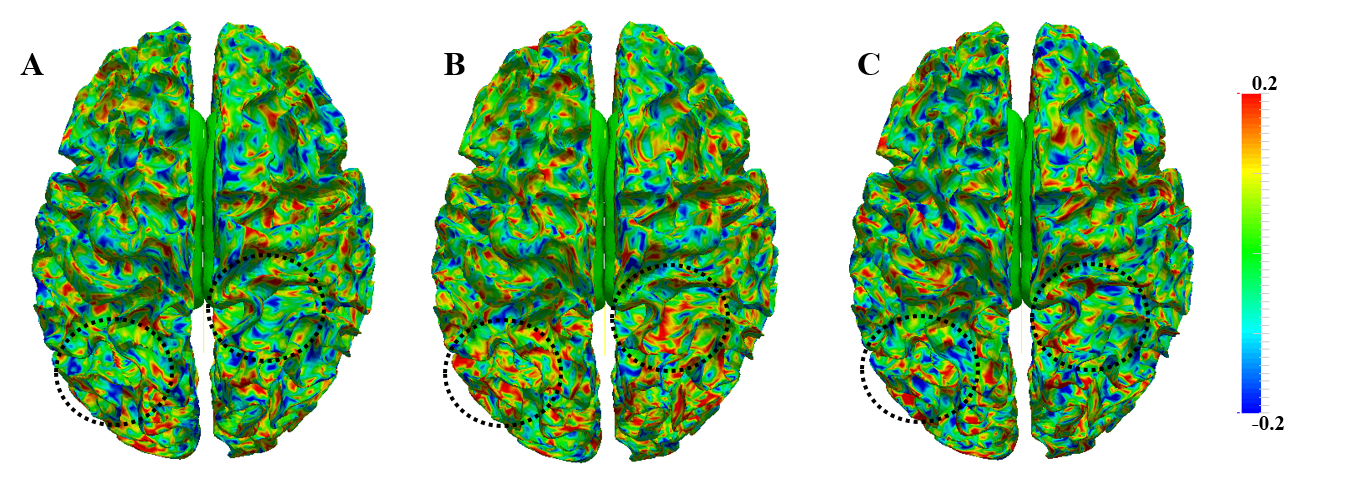


**Supplementary Figure 14.** Illustration of the temporal variability mapped on cortex with different time windows in one subject. (A)-(C) represent the signal segment correlation between three different time window pairs, respectively. Certain example correlation differences among different time window pairs are highlighted by black dashed ovals.
